# Ossification of the posterior capitellum: description of a new pathology and its radiological appearance

**DOI:** 10.1007/s00402-022-04689-7

**Published:** 2022-11-18

**Authors:** M. M. Schneider, B. Hollinger, A. Zimmerer, R. Nietschke, H. Michaely, F. Migliorini, N. Maffulli, K. J. Burkhart

**Affiliations:** 1 Arcus Sportklinik, Rastatter Str. 17-19, 75179 Pforzheim, Germany; 2 University Witten/Herdecke, Witten, Germany; 3 Abteilung Sportorthopädie, Orthopädische Klinik Markgröningen, Markgröningen, Germany; 4 Department of Orthopedics and Orthopedic Surgery, University Medicine Greifswald, Greifswald, Germany; 5 MVZ Radiologie Karlsruhe, Karlsruhe, Germany; 6 Department of Orthopaedic, Trauma, and Reconstructive Surgery, RWTH University Hospital, Aachen, Germany; 7 Department of Medicine, Surgery and Dentistry, University of Salerno, Baronissi, SA Italy; 8 Faculty of Medicine, School of Pharmacy and Bioengineering, Keele University, Stoke on Trent, England, UK; 9 Barts and the London School of Medicine and Dentistry, Centre for Sports and Exercise Medicine, Mile End Hospital, Queen Mary University of London, London, England, UK; 10 University of Cologne, Cologne, Germany

**Keywords:** Sports injuries, MR imaging, Boxing injuries, Handball goalkeeper, Goalie, Boxer, Exostosis

## Abstract

**Background:**

Boxer elbow and handball goalkeeper elbow are causes of impingement characterized by osteophytes formation at the olecranon and coronoid tip as well as their corresponding fossae. Herein, we present another distinct pathology in these patients: the formation of an exostosis at the posterolateral aspect of the elbow.

**Methods:**

Between April 2016 and May 2020, 12 athletes with boxer elbow and handball goalkeeper elbow (mean age of 22 years) suffering from elbow pain were enrolled in the present study. Plain radiography, magnetic resonance imaging (MRI), and computer tomography (CT) scans were used to evaluate the bone conformation of the posterolateral aspect of the elbow. Assessment and staging of the ossification was performed by two independent fellowship-trained elbow surgeons.

**Results:**

Bone marrow edema of the posterior aspect of the elbow at the origin of the anconeus muscle was initially detected in MRI scans. With the progression of the condition, imaging revealed an ossification posterior to the capitellum with bony bridges. In the advanced stage of the disease, the exostoses was unstable as the ossification had no adherence to the posterior capitellum during surgical excision. Plain radiographs are limited in their ability to detect the condition, whereas MRI and CT scans allow to identify a signal enhancement at the posterolateral aspect of the elbow.

**Conclusion:**

In patients without history of elbow trauma, bony irregularities of the posterior aspect of the capitellum may indicate ossification of the posterolateral aspect of the elbow, most likely caused by repetitive hyperextensions.

## Introduction

Elbow injuries in overhead athletes are common [1]. Up to 75% of handball goalkeepers complain of elbow pain [2]. In boxers, elbow injuries are less common, with an incidence of approximately 1–3% [3, 4]. The terms boxer elbow [5–7] and handball (goalkeeper) elbow [2, 8] are common in these sports with similar pathophysiology and the clinical presentation [2, 5–7]. Repetitive hyperextensions lead to internal impingement with the development of osteophytes, mostly the olecranon, less often at the coronoid (hyperflexion) process and at their respective fossae [6, 9]. This causes pain and restricts the range of motion [6, 9]. Magnetic resonance imaging (MRI) is often used for the detection of various elbow pathologies and offers a sufficient evaluation of most changes in the elbow joint. Structural damages of the capitellum involve osteochondritis dissecans, Panner’s disease, fractures, Osborne–Cotterill lesions (OCLs), loose bodies, degenerative changes such as osteophytes, and impaction injuries [10] that have to be distinguished from an MRI phenomenon called “pseudodefect of the posterior capitellum” [11].

We have encountered an abnormal bone formation of the posterolateral aspect of the elbow in handball goalkeepers and boxers, which caused severe pain and restriction in their respective sports. To the best of our knowledge, ossification or exostosis in this area of the elbow has not been previously described. In this clinical investigation, we attempt to characterize such osteophytes formation of the elbow.

## Materials and methods

This study followed the Strengthening the Reporting of Observational Studies in Epidemiology: the STROBE guidelines [12]. This study was performed following the ethical standards of the Declaration of Helsinki. All patients included in this study were aware of the investigation and gave written informed consent. The present study was approved by the Ethic Commission of the Landesaerztekammer Baden-Wuerttemberg, Germany.

### Patient recruitment and study protocol

The retrospective study with prospectively collected data was conducted at the Arcus Sportklinik, Germany between April 2016 and May 2020. All patients who presented an ossification of the posterior capitellum were included. Patients reported of severe pain and a restriction in range of motion, which did not allow them to perform their sports anymore. The inclusion criteria were: (1) athletes involved in overhead sports (e.g., handball, volley ball, tennis, badminton, etc.) and boxing, (2) age 16 to 50 years, and (3) clinical and imaging evidence of an ossification at the posterolateral aspect of the elbow. The exclusion criteria were: (1) prior elbow surgery, (2) pain onset after a distinct trauma, (3) axial deformities of the elbow, (4) uncontrolled disease, such as diabetes or infections, (5) neoplasm, and (6) advanced elbow osteoarthritis.

New bone formation of the elbow was evaluated using different imaging modalities, including plain radiographs, MRI, and CT sequences. The presence of bone marrow edema, bone spur formation, adherence of the ossification to the underlying bone (stable vs. unstable), and its general localization were also investigated. Patient charts and histories were analyzed for patient demographics and prospectively collected. Histological examination was performed in three patients after resection of the ossification.

### Imaging

To detect exostosis of the posterior capitellum, anteroposterior and lateral elbow radiographs as well as MRI and CT were analyzed. Radiographic imaging was performed at various institutions and is therefore heterogenic. Evaluation of all images was performed with the DICOM viewer Horos (v. 3.3.5., Horos Project [13]). The analysis of MRI scans and disease staging was performed independently by two elbow fellowship-trained surgeons for all the included patients.

### Validation and statistics

Data were analyzed using XLSTAT statistics software (Addinsoft, Paris, France). Continuous variables were presented as mean value, and categorical variables were presented as frequency and percentage. Statistical reliability testing of the interpretation of the radiological stage of the disease was performed with intraclass correlation values interpreted as follows: greater than 0.75 = excellent, 0.40–0.75 = fair to good, and less than 0.40 = poor.

## Results

### Patient demographic

The athletes were either handball goalkeepers (*n* = 10) or boxers (*n* = 2), with a mean age of 22 years (range 16–42 years) at first presentation. All patients were right-handed, and the dominant arm was affected in 58% of patients (*n* = 7/12). Handball goalkeepers suffered from slow-onset (range 2–24 months) posterolateral elbow pain lasting for a mean of 9.5 months. Clinical examination revealed tenderness on palpation over the posterolateral aspect of the elbow at the origin of the anconeus muscle. The clinical findings were quite similar to those of synovial fringe syndrome, which also causes pain at the posterolateral aspect of the elbow but slightly more distally. Differentiation between the two conditions may be challenging, but seems possible in most cases because ossification is palpable and painful on deep palpation. However, when ossification is not palpable, distinction is difficult. Nine of the ten handball goalkeepers had a mean restriction of elbow extension of 7°. The symptoms in the two boxers were similar, with tenderness on firm palpation over the posterior capitellum causing the typical pain. However, additional pain during terminal flexion and extension, as previously described for Boxers’ elbow, was noted.

### Syntheses of results

At the first visit, three patients presented with anteroposterior and lateral radiographs. MRI examination was performed in all 12 patients, while seven patients also underwent CT scans (Table 1).

**Table 1:** Distribution of the different imaging techniques for radiographic evaluation

Total no. of patients	Radiographs	MRI	CT
12	3	12	7

### Plain radiography

**Fig. 1:**
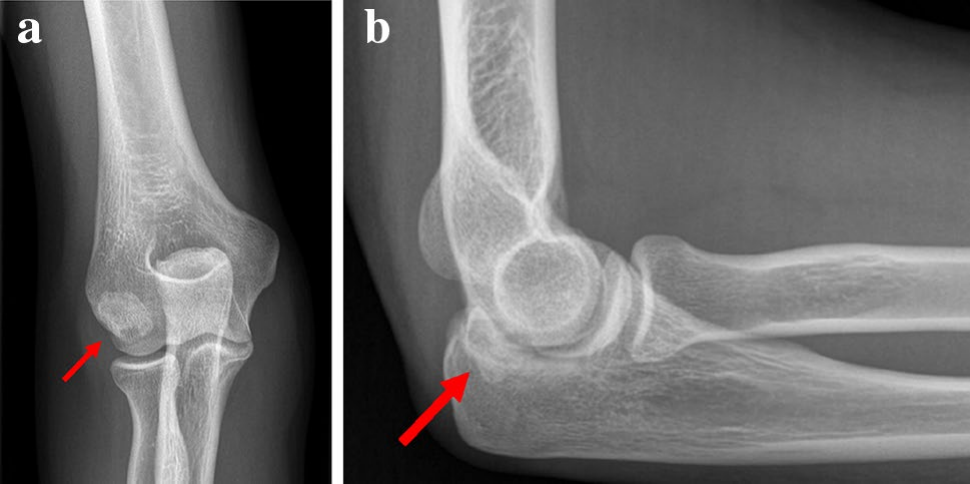
**a** Anteroposterior radiograph of the right elbow of patient no. 10 at first presentation (the arrow marks the new bone formation). **b** Lateral radiograph of the right elbow of patient no. 10 at first presentation (the arrow marks the ossification)

Plain radiographs allowed a restricted assessment of the pathology, as bony changes could only be identified if large ossifications were present (Fig. 1a, b). Plain radiographs are nevertheless, part of the routine diagnostic imaging pathway for elbow pain.

We did not observe any other abnormalities in the plain radiographs of the handball goalkeepers. In contrast, in the boxers’ elbows, osteophytes of the coronoid and olecranon process might be visible on the lateral radiographs. Unfortunately, neither of the two boxers in our series had plain radiographs available, and we did not perform radiographs when MRI was available.

### Magnetic resonance imaging

**Fig. 2:**
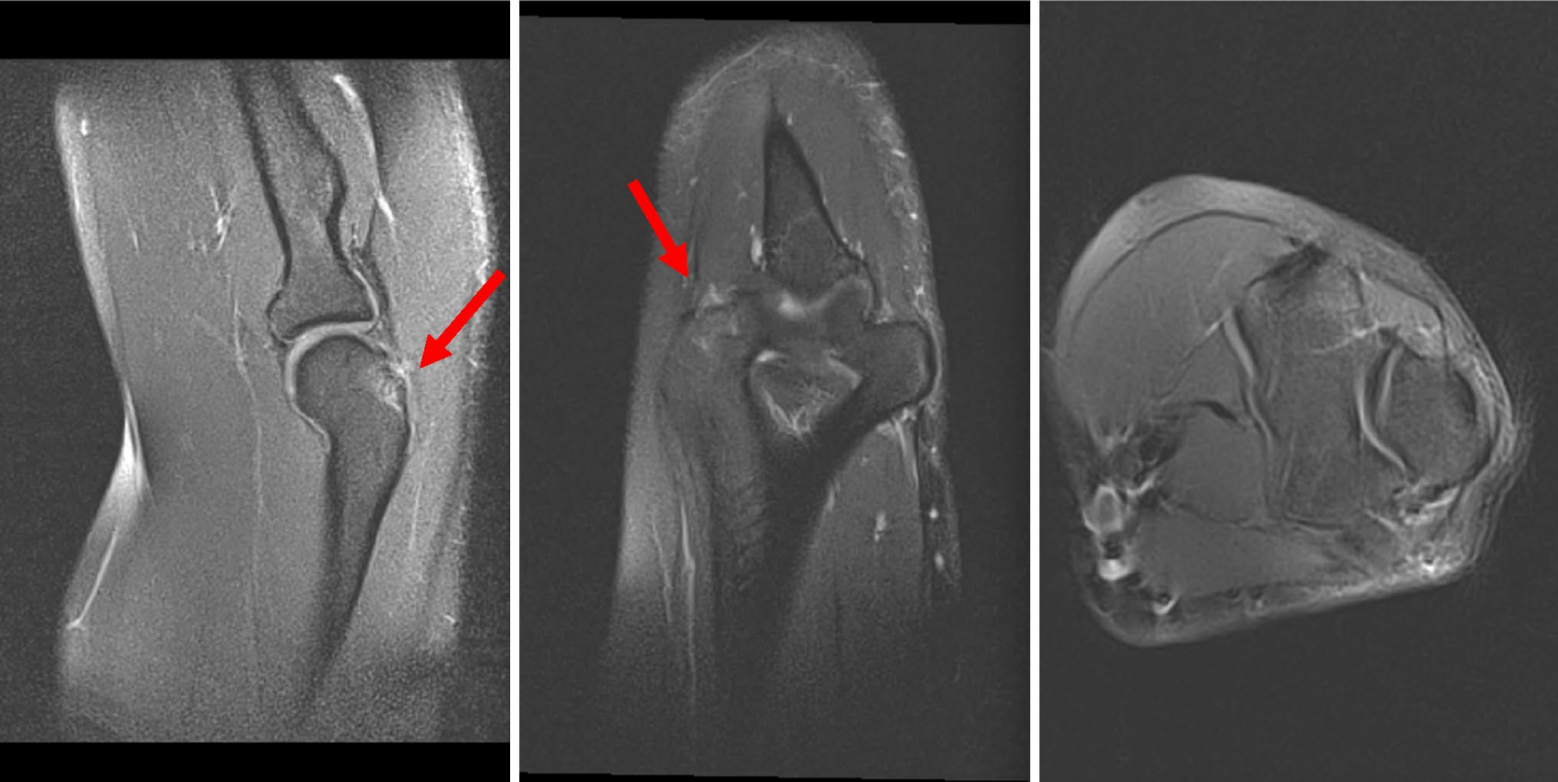
MRI of the right elbow of patient no. 5 at first presentation (the arrow marks the bone marrow edema at the posterior aspect of the elbow)

Changes in the posterior capitellum were best recognized in the sagittal plane view of PD-weighted sequences. Details of the clinical and imaging evaluations of all patients are reported in Table 2. One handball goalkeeper showed signal enhancement of the cancellous bone posterior to the capitellum (Fig. 2).

**Table 2:** Details of the clinical data and MR sequence parameters

Patient	Age	Sex	Sport	Duration of symptoms (months)	Stage	Treatment	Magnetic field strength (Tesla)	Slice thickness (mm)	Field of view (mm * mm)	In-plane resolution (mm * mm)	Sequence type	Echo time (ms)	Repetition time (ms)
1	42	m	Handball goalkeeper	1	III	Surgery	1.5	3	137 * 180	0.47 * 0.74	T2_tse_sag	81	3590
2	20	m	Boxer	24	III	Surgery	1.5	3	150 * 150	0.33 * 0.67	pd_fs_tse_sag	36	3490
3	18	m	Boxer	3	III	Surgery	1.5	3	159 * 159	0.41 * 0.75	pd_fs_tse_sag	29	4310
4	20	m	Handball goalkeeper	12	II	Surgery	1.5	3	120 * 120	0.65 * 0.85	T1_sag_FSAT	12	499
5	17	m	Handball goalkeeper	2	I	Conservative	1.5	3	150 * 150	0.47 * 0.47	PD_tseB_sag_fs	54	4340
6	22	m	Handball goalkeeper	10	III	Surgery	n/a	3	160 * 160	0.5 * 0.5	PD_tseB_sag_fs	41	2480
7	22	m	Handball goalkeeper	12	III	Surgery	3.0	2.5	140 * 140	0.36 * 0.49	pd_fs_tse_sag	34	3000
8	23	m	Handball goalkeeper	12	III	Surgery	1.5	3	150 * 150	0.67 * 0.88	ePD_sag_mSPIR CLEAR	25	2200
9	22	m	Handball goalkeeper	5	III	Surgery	1.5	3	170 * 170	0.53 * 0.53	Pd_tseB_sfs_sag	41	2800
10	18	f	Handball goalkeeper	1	III	Surgery	1.5	3	137 * 180	0.47 * 0.74	T2_tse_sag	81	3140
11	16	m	Handball goalkeeper	4	III	Surgery	1.5	3	137 * 159	0.41 * 0.64	Pd_tse_fs_sag	41	3630
12	22	m	Handball goalkeeper	12	III	Surgery	1.5	3	180 * 180	0.67 * 0.97	ePD_sag_mSPIR CLEAR	25	2200

In our series, the handball goalkeepers had no other abnormalities on MRI. However, the two boxers showed signs of internal impingement with osteophytes on the olecranon and coronoid process and arthrofibrosis in the fossa olecrani. Nonetheless, the two boxers presented because of pain at the posterolateral aspect of the elbow, which could be triggered with pressure. None of the patients revealed ligamentous injuries or signs of elbow dislocation, neither clinically nor during surgery.

### Computer tomography

**Fig. 3:**
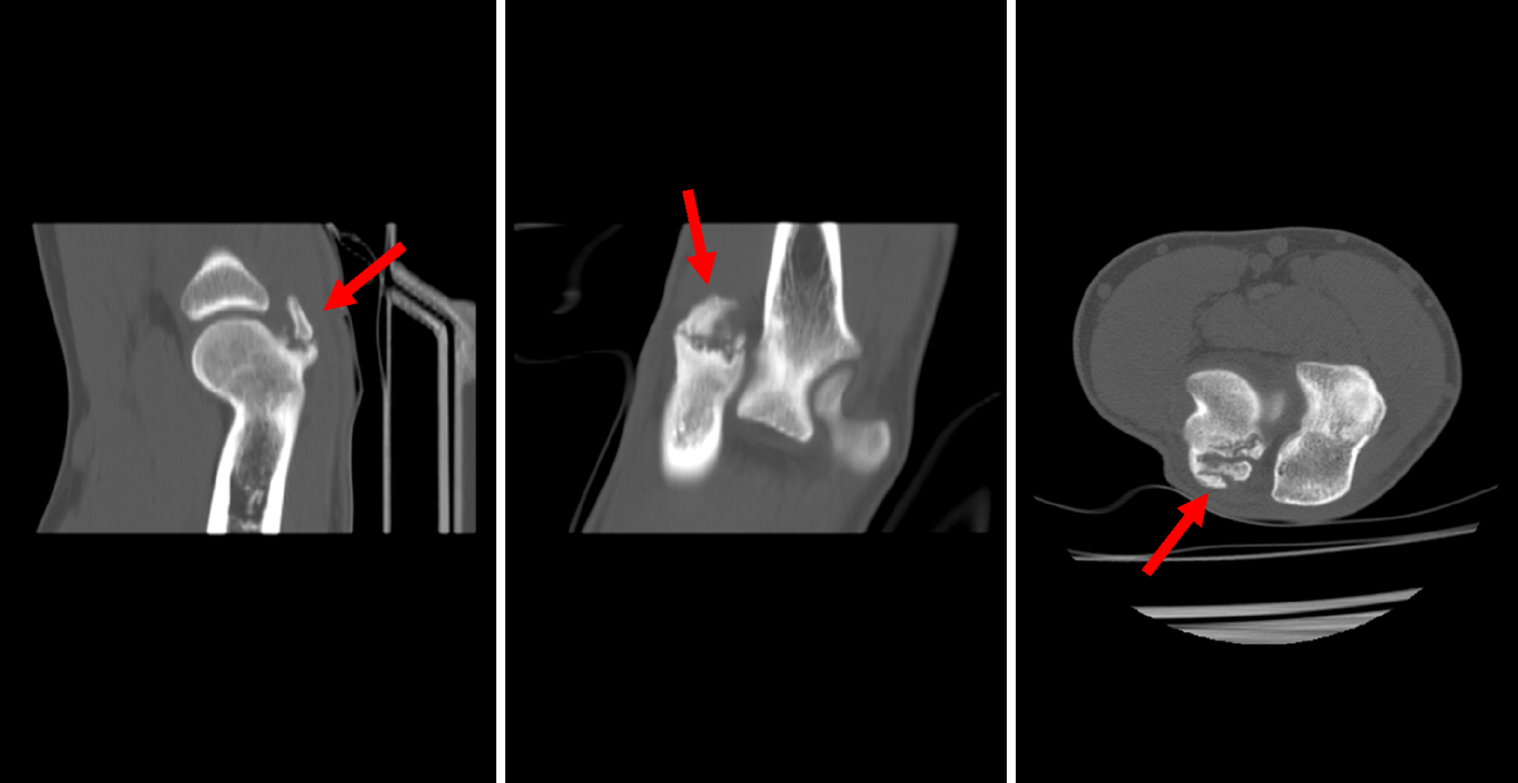
CT of the right elbow of patient no. 7 at first presentation (the arrow marks the ossification)

CT scans were performed in seven patients (Fig. 3). Six of them underwent CT scans before presentation to our clinic. In one patient, we requested an additional CT scan to determine the size and stage of the ossification.

**Fig. 4:**
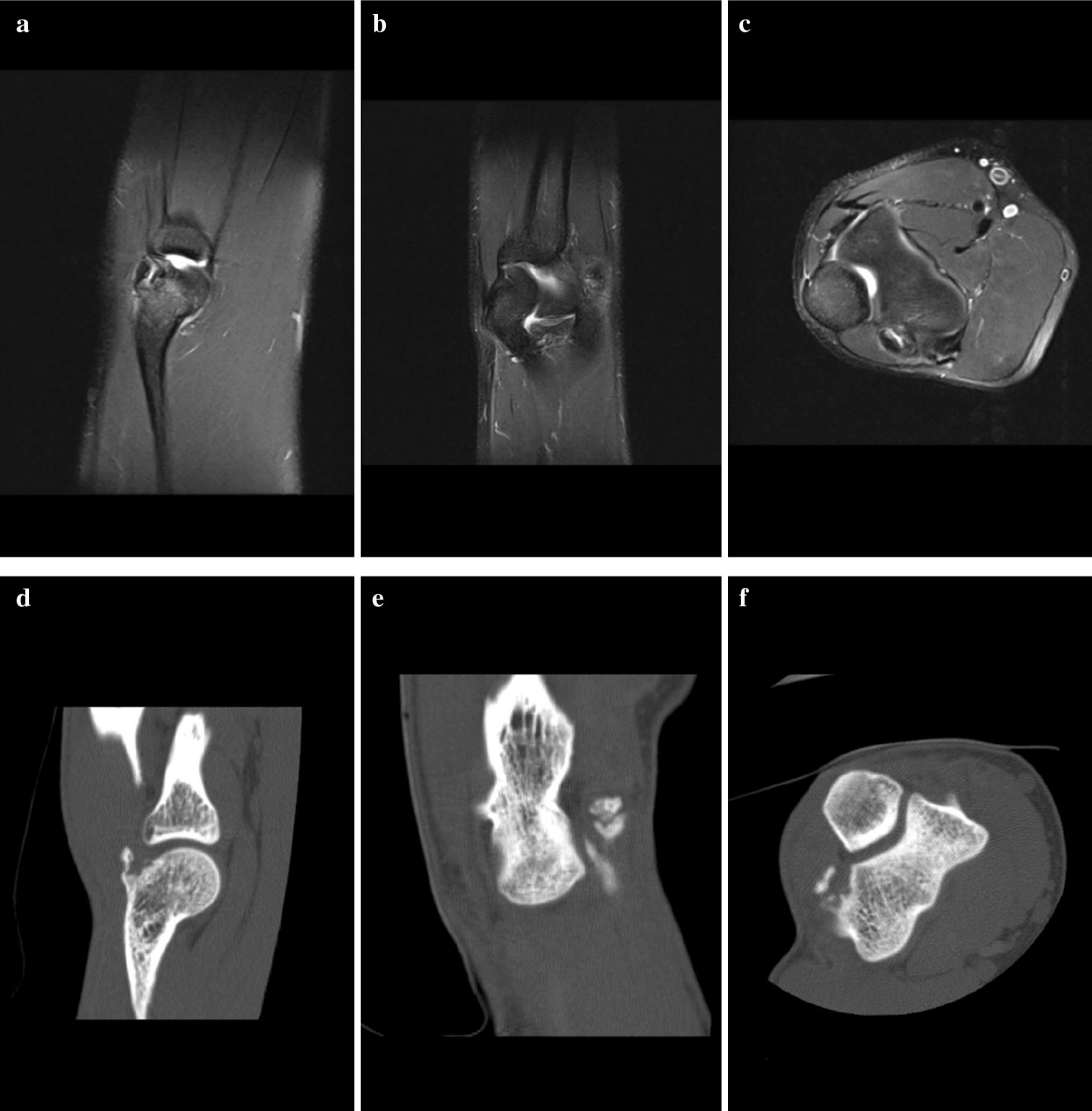
Comparison of MR images (**a**–**c**) and CT scans (**d**–**f**) of the right elbow of patient no. 11

With the experience of 12 cases, CT does provide more information than MRI (size of the ossification, bony conjunction, etc.), especially with 3D reconstructions (Fig. 4a–f). The presence of an ossification and symptoms in our series of patients suggests that conservative treatment is not indicated. Therefore, we would not recommend CT scans in these mostly young handball goalkeepers when the diagnosis was clearly made with the help of MRI scans. Even for the evaluation of the characteristic osteophytes of boxers, MRI might be sufficient in most cases.

In both MRI and CT, measurement of the ossification was difficult, given the size and variety of shapes. However, the exact size of the ossification did not influence our treatment algorithm.

Nonetheless, in the assessment of the ossifications, CT allowed for the most detailed evaluation, enabling the detection of small lesions, which may not be recognized on MRI scans when signal enhancement of the cancellous bone is missing. The sagittal images displayed the new bone formation best.

### Stages

Based on 12 MRI images and seven CT scans, we detected three different appearances that tempted us to divide the exostoses into three stages (Table 3). The intraobserver reliability of the staging of the ossification between two observers on MRI scans was 0.915 (95% confidence interval [CI], 0.867–0.976).

**Table 3:** Staging of exostoses of the posterior capitellum based on MRI/CT findings

Stage		Radiological findings
I	Prodromal	Signal enhancement of the posterolateral aspect of the elbow on MRI (Fig. 2) No ossification
II	Stable ossification	Signal enhancement Ossification (presumable) with connection to the posterior capitellum (Fig. 5)
III	Unstable ossification	Separation of the ossification of the posterior capitellum (Fig. 6)

**Fig. 5:**
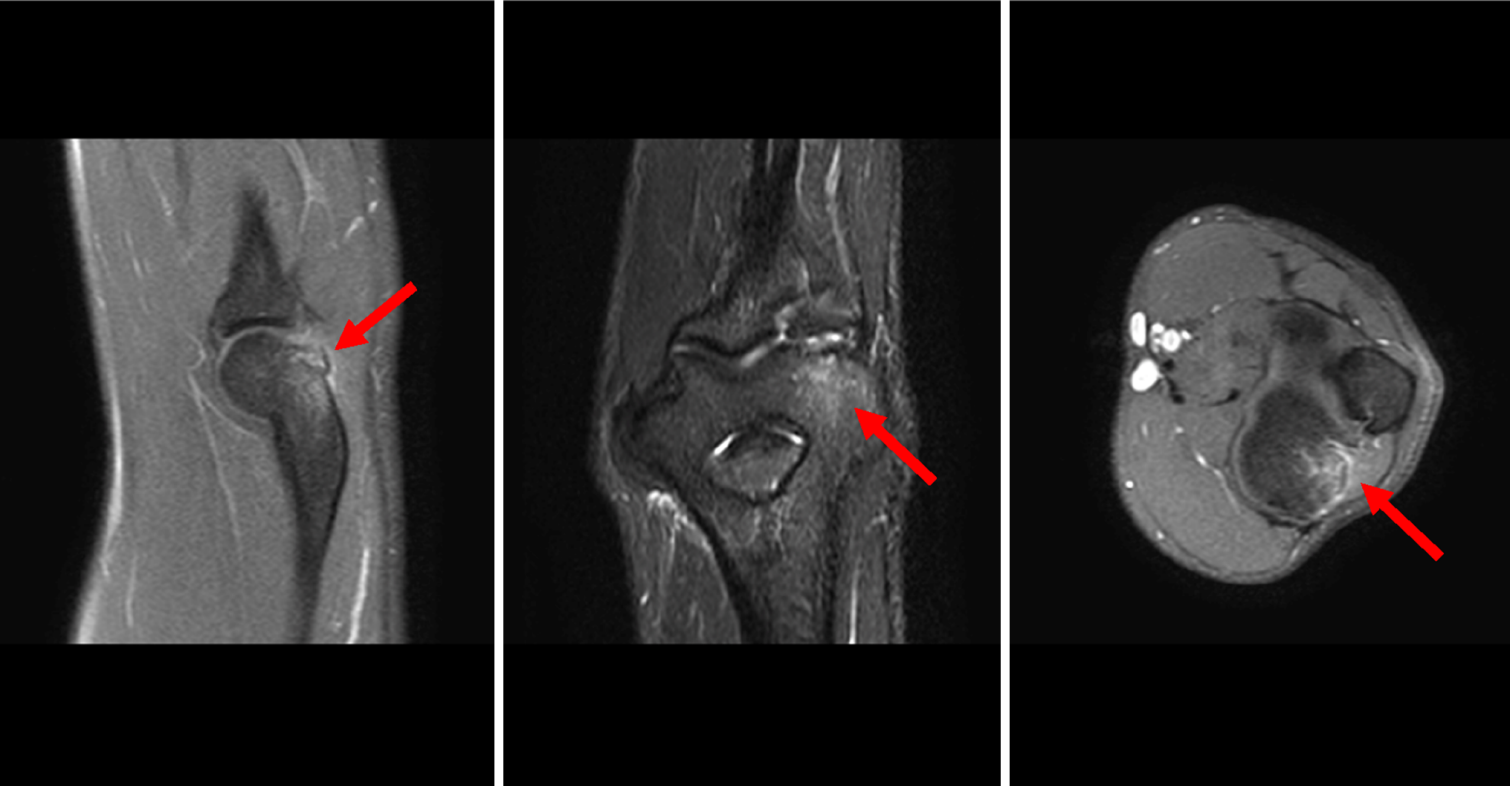
MR images of the right elbow of patient no. 4 at first presentation (the arrow marks the ossification)

**Fig. 6:**
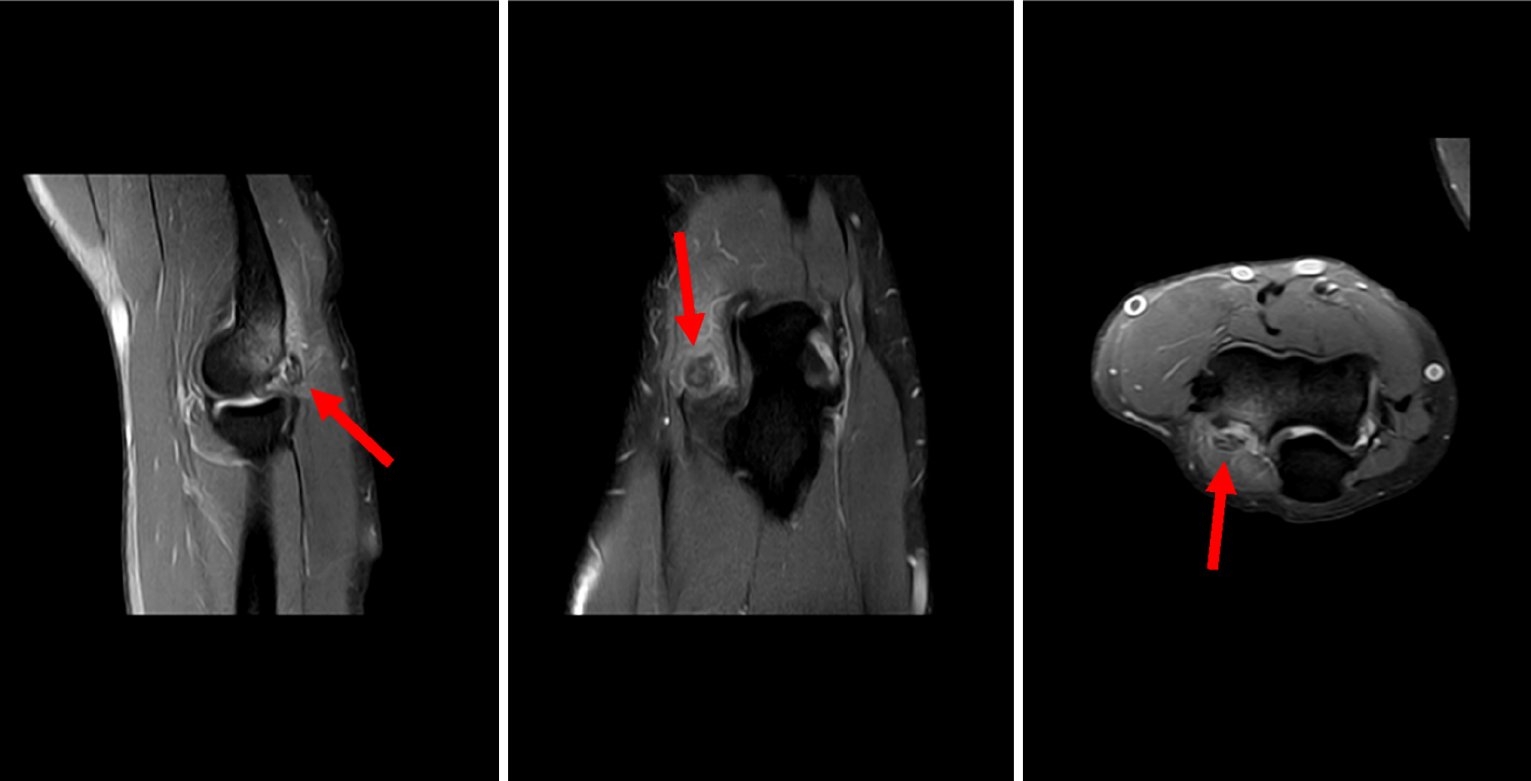
MR images of the right elbow of patient no. 8 at first presentation (the arrow marks the signal enhancement at the posterior aspect of the elbow)

### Management

**Fig. 7:**
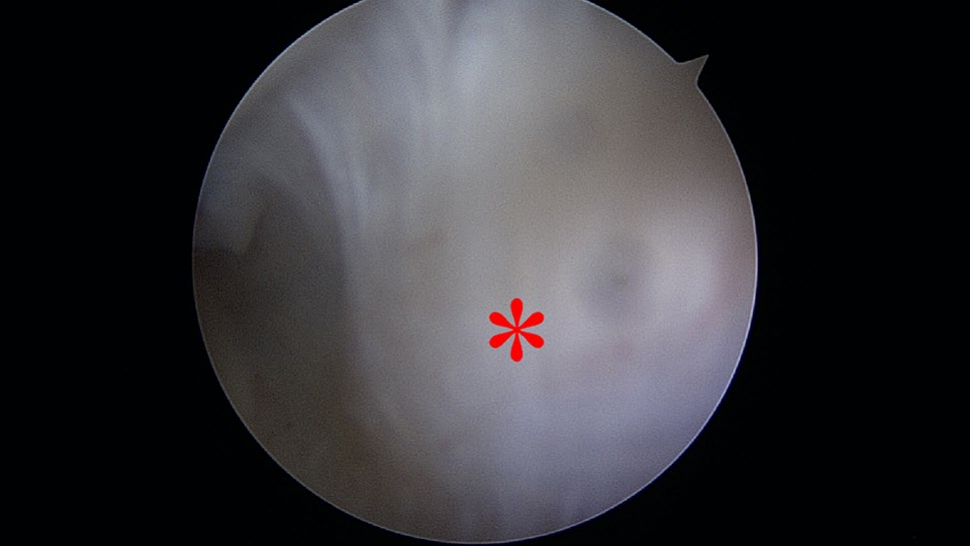
Arthroscopic view from the posterior compartment: toward the radial head, exostosis is visible (marked with an asterisk)

One patient (handball goalkeeper) was treated nonoperatively (stage I). MRI only displayed signal enhancement at the posterior capitellum without exostosis (Fig. 2). The patient received an extension-limiting elbow brace and returned to sporting activities 6 weeks after conservative treatment. The brace was used in training but not in games. All other patients underwent open resection of the new bone formation, with prior arthroscopy in eight cases, to exclude (handball goalkeepers) or treat (boxers) additional intraarticular pathologies. None of the handball goalkeepers had any relevant intraarticular pathologies in addition to a hypertrophic posterolateral synovial fringe. Therefore, preceding arthroscopy might not be necessary when MRI does not display any other abnormalities, and patients are not suffering from relevant loss of motion. In one patient, the ossification was visible during arthroscopy at the posterolateral aspect of the elbow. The ossification was extraarticular, the joint capsule was intact (Fig. 7). In the other cases, the ossification was not visible during arthroscopy.

**Fig. 8:**
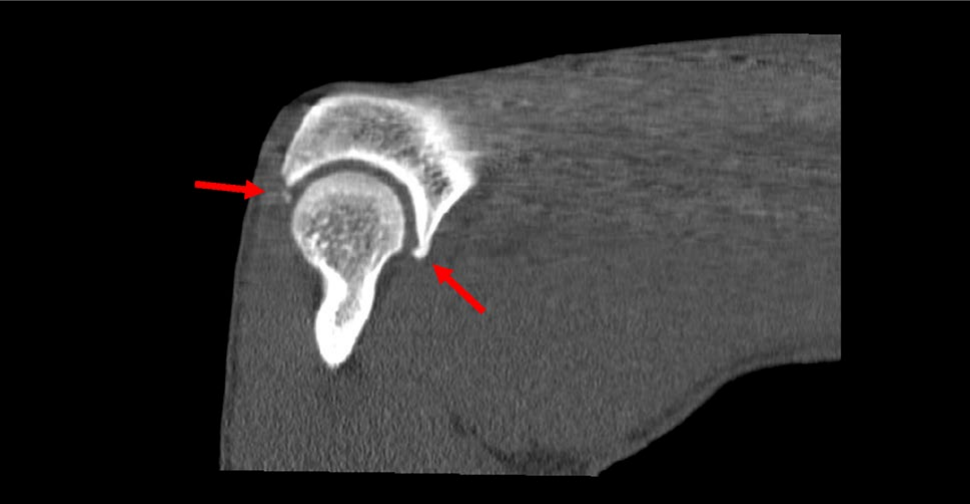
CT scan of the right elbow of patient no. 2 (boxer) displaying osteophytes at the tip of the olecranon as well as the coronoid process (sagittal plane)

Arthroscopy was helpful in both boxers. We performed arthroscopic arthrolysis with resection of the osteophytes at the olecranon and coronoid process and debridement of the fossae (Fig. 8).

MRI showed these changes prior to surgery as well. All patients regained full range of motion after surgery and were able to fully extend their elbow. Therefore, ossification at the origin of the anconeus muscle was most likely the reason for the preoperative deficit in extension in the handball goalkeepers.

### Histology

In three patients, the ossification was sent to a pathology department for further testing without mentioning the suspected diagnosis. In one patient, histological examination revealed a dense connective tissue and an insertional tendinopathy. In the other two histological assessments, the findings indicated an ossification with the recommendation of further imaging evaluation to exclude multiple hereditary exostoses. Because of missing symptoms, we did not perform any additional radiographic evaluations of other joints because of the patients’ age.

## Discussion

The elbow consists of three separate joints surrounded by a single capsule: the humeroulnar, radioulnar, and radiocapitellar joints. The capitellum is ellipsoid with a height between 18 and 30 mm and a width between 9 and 28 mm [14–16]. The posterolateral aspect of the elbow marks the origin of the anconeus muscle. Its function is extension of the forearm, and it contributes to posterolateral stability [17, 18]. In our study, ossification was detected extraarticularly in all surgeries right underneath the origin of the anconeus muscle. The influence of its origin on the development of new bone formation remains unclear. We assume that the specific sequences of movements contribute to the development of ossifications in boxers and handball goalkeepers.

A boxer’s punch consists of sharp forceful extension of the pronated elbow joint. Hitting with maximum power may cause hyperextension, especially when the opponent is missed. Repetition during training and competition might lead to the development of osteophytes of the olecranon, especially the olecranon process [5, 19]. Robinson et al. described an additional anterior impingement that is most likely induced “[…] during ‘clinch’ or push-off maneuvers”, leading to osseous changes in the coronoid process [6]. Handball players can be divided into field players and goalkeepers. Goalies block high-velocity shots with an extended elbow in neutral rotation and an additional valgus force [20, 21]. We have no explanation why this pathology has not been described before, and why in our series only handball goalkeepers and boxers were affected. Most likely, different patterns of muscle contraction/activation in other sports, such as tennis or baseball, may help to prevent uncontrolled hyperextension of the elbow.

The terms “Boxer’s Elbow” and “Handball Goalie’s Elbow” have previously been mentioned in the literature. Osseous changes in the elbows of boxers were first described in 1976. Grenier et al. presented radiographs of two athletes suffering primarily from osteophytes of the olecranon process and loose bodies in the posterior compartment of the elbow. In both cases, the jabbing arm was affected, which point to repetitive hyperextension being the reason for the pathology [5]. Valkering performed surgery in five boxers with posterior elbow impingement. The resection of olecranon osteophytes helped to restore the range of motion, enabling the athletes to return to their previous level of boxing [7]. Robinson et al. published their clinical and radiological findings in seven boxers. In addition to the osteophytes of the olecranon tip, they also found osteophytes of the coronoid process as well as the corresponding fossae, emphasizing the need to evaluate both the anterior and posterior compartments of the elbow during arthroscopy [6]. Tyrdal et al. investigated injuries and subsequent changes in handballers’ elbows [2, 20].

We describe a seemingly new pathology of the elbow in handball goalkeepers and boxers in our series, which may cause pain and restriction in range of motion. Boxers’ and handball goalkeepers’ elbow 2.0 involves an exostosis of the posterolateral aspect of the elbow at the origin of the anconeus muscle, most likely from repetitive hyperextension (boxers) (Fig. 8) and/or combined hyperextension with valgus forces when blocking shots (handball goalkeepers). The exact pathogenesis remains unclear. None of the patients reported an actual trauma or event as the cause of their symptoms. A traction tendinopathy or traction spur might explain the ossification but neither, to our knowledge, have been described at this location. Our suggested stages of the disease are based on 12 patients and their medical imaging and might be modified as more cases accumulate.

Different articles have described abnormalities of the posterior capitellum. One such abnormality is the pseudodefect of the capitellum [11, 22]. The posterior part of the capitellum is not covered by cartilage. The usually prominent notch at the junction between the posterior capitellum and the humerus should be distinguished from other pathologies, such as dislocation or at least subluxation, leading to an impaction injury [23, 24]. The pseudodefect was easy to discriminate from the posterior ossification.

Other differential diagnoses are OCLs, intraarticular loose bodies, osteophytes in elbow arthritis, traction tendinopathy, myositis ossificans and impaction injuries. OCLs are bony defects at the posterolateral corner of the capitellum, very close to where we found the ossifications. We are aware that the signal enhancement of the posterior capitellum (stage I) that we mentioned in our series might be confused with an OCL. However, we believe that these are different entities for several reasons, and the risk of confusion might only be an issue in stage I (not in stages II or III). First, an OCL is normally consequent to a trauma. Every patient was asked multiple times whether he experienced dislocation or subluxation of his elbow. None recalled such an event, which makes OCLs unlikely. Second, in our patients, pain developed slowly. Dislocation or impaction of the capitellum causes sudden and immediate pain. Third, we believe that we observed the development of the pathology from initial bone edema to lose extra-articular ossification, although we cannot state that fact since we do not have repeated longitudinal imaging to our patients. Successful conservative treatment with an extension limiting elbow orthesis makes OCL doubtful as well. Foremost, none of the patients exhibited signs of posterolateral rotatory instability or ligamentous injuries at clinical or arthroscopic evaluations, as described in patients suffering from OCLs [23, 25, 26] or in corresponding lesions at the radial head. OCLs leave a groove at the capitellum, which was not detected in any of our patients. In addition, elbow dislocation in boxers and handball goalkeepers is rare.

Elbow arthritis can cause the development of osteophytes and loose bodies. However, both occur intra-articularly. In our series, all ossifications were extra-articular. During open surgery, the location of the ossification was always right under the origin of the anconeus muscle, which was elevated for resection of the ossification. The joint capsule was intact in all patients. In our series, the mentioned osteophytes might have contributed to the pain of both boxers. We believe that their main source of the complaint was the exostosis, since preoperative pain was described at the posterior capitellum.

An ossification posterior to the capitellum might be easily overlooked in plain radiographs. We encourage prompt MRI examinations in athletes with elbow pain. MRI is the modality of choice to detect cartilage and/or ligament abnormalities of the elbow joint. Especially in boxers and handball goalkeepers with posterolateral elbow pain, MRI offers essential details. It helps to detect ossifications at the posterolateral capitellum at an early stage, although it might not allow for discrimination of stable and unstable lesions. Early detection might help to identify patients at an early stage of the disease in whom surgery might be prevented with conservative measures.

The patient with no ossification but a sole signal enhancement (stage I) reported pain over a period of only two months (Fig. 2), which represents a significantly shorter duration of symptoms than the other patients (average eight months). Early clinical and imaging examinations might have helped to detect the condition at its initial phase. On the other hand, ossifications might initially be asymptomatic, since some patients presented soon after their first symptoms but displayed advanced ossification on imaging (patients 1 and 10). In addition to injuries to the medial collateral ligament, osteophytes of the coronoid and olecranon process and their fossae can often be seen in boxers suffering from extension and flexion deficits and typical impingement pain. Discrimination of “stable” and “unstable” ossifications can be difficult on MRI.

We performed surgery when bone formation was visible on MRI. Discrimination between stages II and III was only made on MRI/CT, distinguishing between stable and unstable ossifications. Patients with stage II and stage III condition did not differ clinically both suffering from pain and restriction in their sporting ability. However, most patients presented with stage III ossifications. Conservative treatment failed in two patients. Because of the ongoing pain and the restriction in range of motion, nonsurgical treatment seems not promising in patients with stage II or III ossifications.

Despite the possibility of distinguishing between stage II and stage III, we do not recommend CT scans in these patients since additional value is low. In cases of missing signal enhancement and very small exostoses on MRI, CT scans might help to ensure the diagnosis of an extra-articular ossification of the posterior capitellum. However, the cases are likely uncommon, since most patients presented with a clearly detectable ossification. For inexperienced surgeons, CT scans may offer additional value when treating boxers by providing the precise location of osteophytes at the olecranon and coronoid process. We believe that MR or CT arthrography have no value in this group of athletes since the pathology is not intra- but extra-articular.

In our series, the staging of the disease according to our proposed system showed high intraobserver reliability.

Boxers and handball goalkeepers seem to have an elevated risk of developing this pathology. In addition to male sex (11:1 ratio), no other predisposing factors could be determined. Our presentation of an ossification at the posterolateral aspect of the elbow caused by repetitive hyperextension has several limitations.

Although we are convinced that our hypotheses regarding the pathogenesis (repetitive forceful hyperextension with a valgus and supination load causing the development of a bone bruise and subsequent ossification) and treatment are reasonable, our experience is only based on 12 patients. We therefore do not claim to have fully understand the lesion and might not be able to precisely characterize it, although the clinical and imaging results are promising and suggest that the observed staging and the corresponding treatment recommendations are applicable.

The different appearances observed in MRI/CT and during surgery have not been followed longitudinally. That means we have no radiological proof of the sequence of the three stages. However, following a patient over time without providing sufficient therapy does not seem acceptable.

All patients reported reduced pain, were able to return to their respective sport and were satisfied with the postoperative outcomes. This suggests that our treatment was, and a longitudinal imaging might not be reasonable. The goalkeeper with a sole bone bruise was treated without surgery and the signal enhancement dissolved over time. Furthermore, not every patient received the same medical imaging. The reason for that was that some patients already presented with cross-sectional imaging modalities, while others did not. The more cases we evaluated, the less did an additional computed tomography contribute to clinical decision-making. Therefore, we did not recommend computed tomography in order to prevent radiation exposure. The differentiation between a “stable” and “unstable” lesion had no clinical relevance and was primarily enabled by the surgical findings.

Boxers might have had additional problems caused by intraarticular osteophytes so that the sole extent of improvement caused by the resection of the ossification remains unknown. Finally, although data were collected prospectively, this study was initiated retrospectively, meaning we started evaluating patients after we treated them. Moreover, we did not follow the advice of the pathologists to perform pelvic X-rays to determine the existence of a multiple hereditary exostoses. Our patients did not report of any pelvic or hip pain, and therefore, we did not perform additional imaging. This study was thought to describe the pathology and its radiological appearance and therefore did not include clinical follow-ups after surgery.

With our description, radiologists, especially musculoskeletal radiologists, and orthopedic surgeons should be able to assess changes in the posterolateral corner of the capitellum that cannot be explained by OCLs, elbow dislocation or osteophytes in that region in patients suffering from elbow arthritis. The extent of the osseous changes allows for grading of the disease. If ossification is clearly recognized on MRI, we believe that conservative treatment is not promising because mechanical irritation at the posterolateral capitellum will remain after conservative treatment. However, we did not compare conservative versus operative treatment in patients with manifest abnormalities.

## Conclusion

In patients without history of elbow trauma, bony irregularities of the posterior aspect of the capitellum may indicate ossification of the posterolateral aspect of the elbow, most likely caused by repetitive hyperextensions.
